# Anticancer Properties and Mechanisms of Singly-Protonated Dehydronorcantharidin Silver Coordination Polymer in a Bladder Cancer Model

**DOI:** 10.3389/fphar.2021.618668

**Published:** 2021-02-23

**Authors:** Changkuo Zhou, Ganyu Wang, Weiqiang Jing, Xuejie Tan, Hu Guo

**Affiliations:** ^1^ Department of Urology, Qilu Hospital, Shandong University, Jinan, China; ^2^ Department of Pediatric Surgery, Qilu Hospital, Shandong University, Jinan, China; ^3^ School of Chemistry and Pharmaceutical Engineering, Qilu University of Technology (Shandong Academy of Sciences), Jinan, China

**Keywords:** caspase-3, apoptosis, cell cycle, bladder cancer, Ag-SP-DNC

## Abstract

Bladder cancer is the most common malignant urinary system tumor. Chemotherapy is frequently used as a treatment regimen for patients with bladder cancer, however, new and effective drugs for bladder cancer need to be developed. The present study examined the effects and mechanisms of Ag-SP-DNC, a silver and singly-protonated dehydronorcantharidin complex, on bladder cancer *in vitro* and *in vivo*. It was identified that Ag-SP-DNC suppressed cell proliferation and induced apoptosis in bladder cancer cells *in vitro*, a suppression associated with G0/G1 phase arrest and elevated intracellular reactive oxygen species (ROS) levels. Furthermore, Ag-SP-DNC enhanced the cleaved caspase-3 levels, disrupted the mitochondrial transmembrane potential balance, and induced intracellular calcium overload. The Ag-SP-DNC-induced bladder cancer cell apoptosis was significantly decreased following treatment with a broad caspase inhibitor, zVAD-fmk. In addition, treatment of MB49 tumor-bearing mice with Ag-SP-DNC significantly inhibited tumor growth and decreased the anti-apoptosis and cell cycle promotion protein levels in the tumor. The results of the present study suggested that Ag-SP-DNC elicits a strong anticancer effect against bladder cancer, and can therefore be used as a promising treatment for bladder cancer.

## Introduction

Cancer is a major public health problem worldwide, with bladder cancer being the 4th most commonly diagnosed cancer in men in the United States (Antoni et al., 2017; Siegel et al., 2020). Approximately 70% of bladder cancers are non-muscle-invasive bladder cancers upon initial diagnosis, which usually have a relatively favorable prognosis but are associated with considerable morbidity and a high treatment cost (Chang et al., 2016; Robertson et al., 2018). A major aim in the treatment of bladder cancer is the prevention of tumor recurrence and progression. Treatment regimens for bladder cancer and their efficacy vary depending on disease stage, grade and associated risk factors (Alifrangis et al., 2019). The standard treatment for patients with an intermediate-to-high risk of recurrence consists of intravesical administration of agents including Bacillus Calmette-Guérin and mitomycin C (Packiam et al., 2017). In the case of muscle-invasive bladder cancer, a current standard of care is cisplatin-based neoadjuvant chemotherapy, followed by radical cystectomy (Plimack et al., 2015).

Chemotherapeutic agents have certain limitations when it comes to treating cancer. The cytotoxicity as well as non-selective nature of chemotherapy result in significant damage to the normal cells and insufficient penetration into the tumors. Nowadays combination therapy has been highly recommended for cancer treatment due to its advantages of reducing dose and decreasing development of drug resistance. Such as natural compounds which possess significant anticancer, pro-oxidant potential and high safety have been utilized as an antitumor agent against several carcinomas throughout history (Martin-Cordero et al., 2012; Pandey et al., 2018). Besides, RNA interference (RNAi) based therapeutic approaches are under exploration to cure cancer currently. Cancer cells lose the ability to regulate apoptosis, thus, transfection anticancer small interfering RNA (siRNA) and micro RNA (miRNA) into cells to knock down the carcinogenic genes by targeting the mRNA expression is possible (Gandhi et al., 2014; Pandey et al., 2019b). Since the development of immunological checkpoints, the programmed cell death protein 1/ligand-1 (PD-1/PD-L1) pathway has emerged as an exciting therapeutic target for patients with advanced bladder cancer (Eckstein et al., 2019). Based on the data from a previous study, the objective response rate of PD-1/PD-L1 inhibitors is ∼20% in all patients (Fan et al., 2019). Developing innovative chemotherapies for bladder cancer with a superior anti-tumor efficacy and reduced side effects continues to be a challenge. In order to further develop the biological efficacy of anticancer drugs, further research for a better curative trial of bladder cancer is essential. The success of platinum-based chemotherapy in the clinic and its tolerance following long-term use have led researchers to find new metal-based complexes for bladder cancer treatment.

**FIGURE 1:**
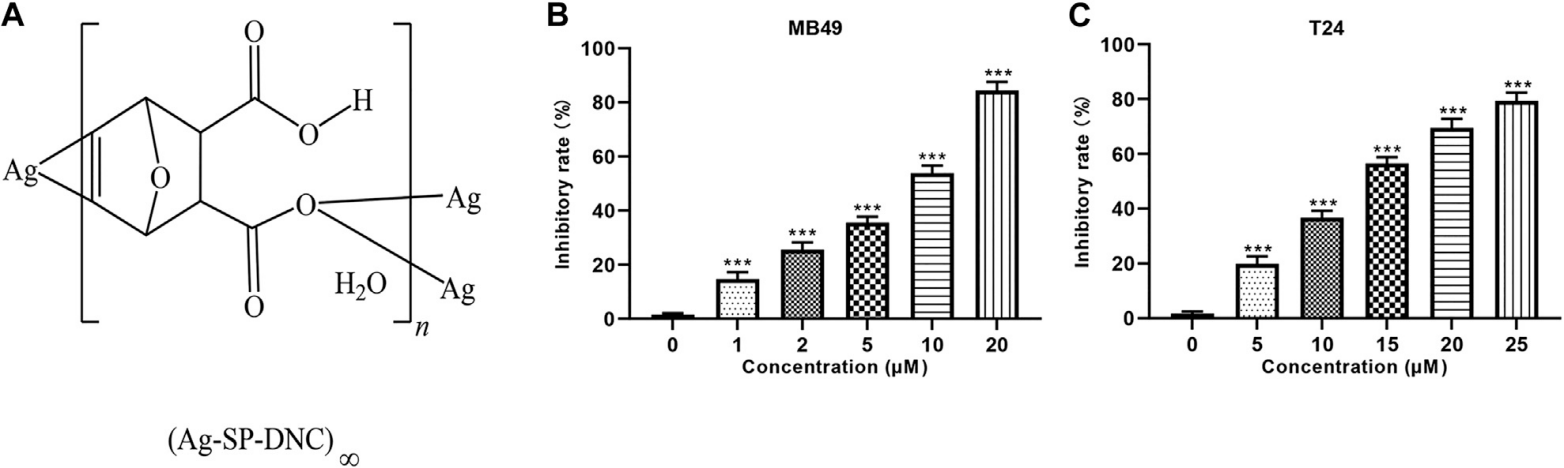
Ag-SP-DNC shows potent growth inhibition in bladder cancer cells *in vitro*. **(A)** Chemical structure of Ag-SP-DNC. **(B)** Inhibitory rate histogram of MB49 treated with Ag-SP-DNC for 48 h. Data are mean ± SEM; n = 3. ***p < 0.001. **(C)** Inhibitory rate histogram of T24 treated with Ag-SP-DNC for 48 h. Data are mean ± SEM; n = 3. ***p < 0.001.

Among the many organometallic compounds with anti-proliferative activities against cancer cells, those based on silver have attracted much attention, as they have interestingly been shown to possess a dual activity, inhibiting the proliferation of tumor cells and inducing apoptosis, while being biocompatible with normal tissue cells (Banti and Hadjikakou, 2013; Durai et al., 2014; Li et al., 2014). The therapeutic properties of silver dates back to Hippocrates, where it was used as an antimicrobial agent to treat extensive burns and chronic ulcers, as well as to prevent conjunctivitis in infant’s eyes, for which it is used until today. As compared with well recognized pharmacological agents, such as antimicrobial agents, silver-based compounds have not yet been well investigated as anti-cancer agents (Morones-Ramirez et al., 2013). In a previous study, Tan et al synthesized a novel silver and singly-protonated dehydronorcantharidin complex, poly[[[μ_3_-(5,6-η):κO^2^: κO^2^-(±)-(1S,2S,3R,4R)-3-carboxy-7-oxabicyclo[2.2.1]hept-5-ene-2-carboxylato]silver(I)] monohydrate] (Ag-SP-DNC; Figure 1A). Previous studies evaluated the anti-proliferative effects of Ag-SP-DNC on human lung cancer cells and mouse colon cancer (Jin et al., 2015). To explore the possibility of Ag-SP-DNC being used for the treatment of bladder cancer, the anticancer effects of Ag-SP-DNC on human bladder cancer cells and a mouse bladder cancer model were investigated. The results indicated that Ag-SP-DNC is effective when used on a bladder cancer model, and the mechanisms underlying the treatment of bladder cancer with Ag-SP-DNC were explored.

## Materials and Methods

### Materials

Ag-SP-DNC was synthesized and provided by Tan et al. RPMI-1640 medium were purchased from MACGENE. Fetal Bovine Serum (FBS) were purchased from Corning. Sulforhodamine B (SRB), RNase A, Propidium Iodide (PI) and rat IgG were purchased from Solarbio. FITC AnnexinV Apoptosis Detection Kit and anti-Ki67-PerCP cy5.5 monoclonal antibody were purchased from BD Bioscience. Triton X-100 solution, Ca^2+^ specific fluorescent probe Fluo-4/AM, JC-1 Staining Kit, Reactive Oxygen Species Assay Kit, GreenNuc^TM^ Caspase-3 assay kit, One Step TUNEL Apoptosis Assay Kit were purchased from Beyotime. Western blot Antibody Diluent was purchased from Epizyme. BCA protein assay kit was purchased from Thermo Fisher Scientific. Western Bright^TM^ ECL-Plus was purchased from EMD Millipore. β-actin(13E5) Rabbit mAb were purchased from Cell Signaling Technology. D-Luciferin sodium salt was purchased from YEASEN.

### Cell Culture

MB49 and T24 cells were cultured in RPMI-1640 medium [cat. no., CM10041; MACGENE (Beijing) Biotechnology Ltd.] supplemented with 10% fetal bovine serum (cat. no., CS002.500; Corning Inc.), 100 U/ml penicillin and 100 μg/ml streptomycin. The cells were maintained in the recommended cell culture media at 37°C in 5% CO_2_. When cells reached 40–50% confluence following plating, the medium was changed with Ag-SP-DNC treatment.

### Sulforhodamine B Assay

The SRB assay was used to determine cell density, based on the measurement of cellular protein content. Briefly, cells were seeded into 96-well plates, and then treated with various concentrations of Ag-SP-DNC for 48 h. The cell monolayers were fixed with 10% trichloroacetic acid and stained with 0.4% SRB in 1% acetic acid. Following washing, the protein-bound dye was dissolved in 10 mM Tris base solution for OD determination at 510 nm using an ELISA plate reader (Vichai and Kirtikara, 2006).

### Ki67 Analysis

Ki67 is a nuclear antigen significantly correlated with cell proliferation. The cells were collected, washed twice in PBS, and then suspended with 4% paraformaldehyde and fixed for ∼30 min. Following washing twice and resuspending with PBS, the single-cell suspension was added to ice-cold methanol, reacting on the ice for 15 min. Next, cells were labeled with pre-diluted anti-Ki67-PerCP cy5.5 monoclonal antibody (cat. no., 561284; BD Biosciences) and incubated for 30 min at room temperature (Kim and Sederstrom, 2015). The samples were measured by flow cytometry, and the proliferative activity was expressed as the fluorescence intensity of Ki67.

### Cell Cycle Detection

Twenty-four hours after seeding cells in a 6-well culture plate, cells were treated with Ag-SP-DNC for 24 h. They were then washed with PBS and fixed with cold 70% ethanol overnight at -20°C. Next, cells were incubated with 200 μg/ml RNase and 50 μg/ml propidium iodide (PI) at 37°C in the dark for 30 min. Cell cycle phase distribution was examined by flow cytometry (Galvin et al., 2019).

### Annexin V-Fluorescein Isothiocyanate/PI Apoptosis Assay

Double staining with Annexin V-FITC and PI was performed using the FITC AnnexinV Apoptosis Detection Kit (cat., no. 556547; BD Biosciences), according to the manufacturer’s instructions. Following treatment with different concentrations of Ag-SP-DNC for 48 h, bladder cancer cells were stained with FITC-Annexin V and PI. Cells were divided into dead, viable and apoptotic cells, and then the relative ratio of apoptotic cells was compared with each group (Yen et al., 2014).

### *In vivo* Mouse Bladder Cancer Model

Male C57 mice aged 6–8 weeks were purchased from the Laboratory Animal Center of the Shandong University. The MB49 cell line with a luciferase-expressing plasmid (Ubi-MCS-firefly_Luciferase-IRES-Puromycin) was provided by Shanghai GeneChem Co., LTD. The mice under anesthetization were injected with fLuc-MB49 cells (1 × 10^6^) into their bladder wall via a 0.33 × 12.7 mm insulin syringe (BD U-100 insulin). The D-Luciferin sodium salt (YEASEN, cat: 40901ES01) was used for the bioluminescence imaging (10 mg/kg). Three days after the cell inoculation, the bioluminescence of bladder could be detected by IVIS Lumina system (Fabris et al., 2012). The drug therapy consisted of an intravenous administration of Ag-SP-DNC (5 and 10 mg/kg) and carboplatin (10 mg/kg). A 5% glucose solution by tail-vein injection was used as the control for drug treatment. Three days post-implantation, tumor-bearing mice were randomized into groups and started drug administration once every 2 days until day 14. All animal experiments were conducted in full accordance with protocols approved by the Ethical Committee of the Qilu Hospital of Shandong University (Jinan, China).

### Histological Analysis

After mice were sacrificed by cervical dislocation, the tissues (heart, liver, spleen, lung and kidney) from mice treated with Carpoplatin and Ag-SP-DNC were dissected and fixed with 4% paraformaldehyde (v/v) in phosphate-buffered saline (PBS) for 48 h. Paraffin-embedded tissues were sectioned with 5-μm thickness. Haematoxylin and eosin (H&E) staining was performed on the sections. The histopathological changes of vital organs were evaluated using light microscopy.

### Western Blot Analysis

Tumor protein lysates were collected in ice-cold RIPA buffer supplemented with a complete protease and phosphatase inhibitor cocktail using a tissue homogenizer. Protein concentration was quantified using a BCA protein assay kit (cat. no., NCI3227CH; Thermo Fisher Scientific, Inc.). The proteins were blotted onto a PVDF membrane, and then the membrane was blocked with 5% non-fat milk in Tris-buffered saline and incubated with a primary antibody overnight at 4°C. Following washing with TBST, the membrane was incubated with a secondary antibody (Bo Zhang et al., 2020). The signal was visualized using an ECL-Plus (cat. no., WBKLS0050; EMD Millipore).

### Detection of Reactive Oxygen Species

A Reactive Oxygen Species Assay Kit (cat. no., S0033; Beyotime) was used to measure intracellular ROS levels (Wang et al., 2020). Briefly, cells treated with Ag-SP-DNC for 24 h were trypsinized, washed twice with ice-cold PBS and incubated with fluorescent dyes for flow cytometric analysis, according to the manufacturer’s instructions. The levels of intracellular ROS were determined by flow cytometry.

### Mitochondrial Membrane Potential Analysis

The mitochondrial membrane potential was detected using a JC-1 mitochondrial membrane potential assay kit (cat. no., C2006; Beyotime Institute of Biotechnology). Briefly, the cells were collected, washed twice in PBS to remove the cell media and incubated with JC-1 staining solution for 20 min at 37°C. The cells were then washed twice with 1x JC-1 buffer and the fluorescence intensity was analyzed using flow cytometry (Perelman et al., 2012).

### Caspase-3 Assay

For the detection of caspase-3 activity, cells were cultured in 6-well plates, treated with Ag-SP-DNC for 48 h and evaluated using a GreenNuc^TM^ Caspase-3 assay kit (cat. no., C1168S; Beyotime Institute of Biotechnology), according to the manufacturer’s instructions. Single-cell suspensions were added in 1 μl GreenNuc^TM^ Caspase-3 substrate, mixed sufficiently and incubated for 20 min at 37°C in the dark (Poczta et al., 2019). The levels of caspase-3 activity were determined by flow cytometry.

### Ca^2+^ Concentration Determination

The cytoplasmic calcium was measured by Fluo-4 AM (cat. no., S1060; Beyotime Institute of Biotechnology). Briefly, cells treated with Ag-SP-DNC for 24 h were trypsinized, washed twice with ice-cold PBS and incubated with calcium probe for the flow cytometric analysis. Intracellular Ca^2+^ concentration was determined by flow cytometry (Duan et al., 2019).

### Terminal Deoxynucleotidyl Transferase dUTP Nick End Labeling (TUNEL) Analysis

TUNEL was used for detection of apoptosis. The collected cells were suspended with 4% paraformaldehyde to fix for 30 min, then washed twice and resuspended with PBS. Cells were permeabilized by Triton X-100, washed twice and mixed sufficiently with TUNEL detection solution for 1 h at 37°C (Majtnerová and Roušar, 2018). The stained cells were then analyzed by flow cytometry.

### Statistical Analysis

Data analysis was performed using Prism software (GraphPad Prism version 7.0; GraphPad Software, Inc.). Statistical analyses were performed using one-way ANOVA. Error bars for SEM are shown. Where indicated in the figures, degrees of *p*-value significance are as follows: *p < 0.05, **p < 0.01 and ***p < 0.001.

## Results

### Ag-SP-DNC Shows Potent Growth Inhibition in Bladder Cancer Cells *in vitro*

**FIGURE 2:**
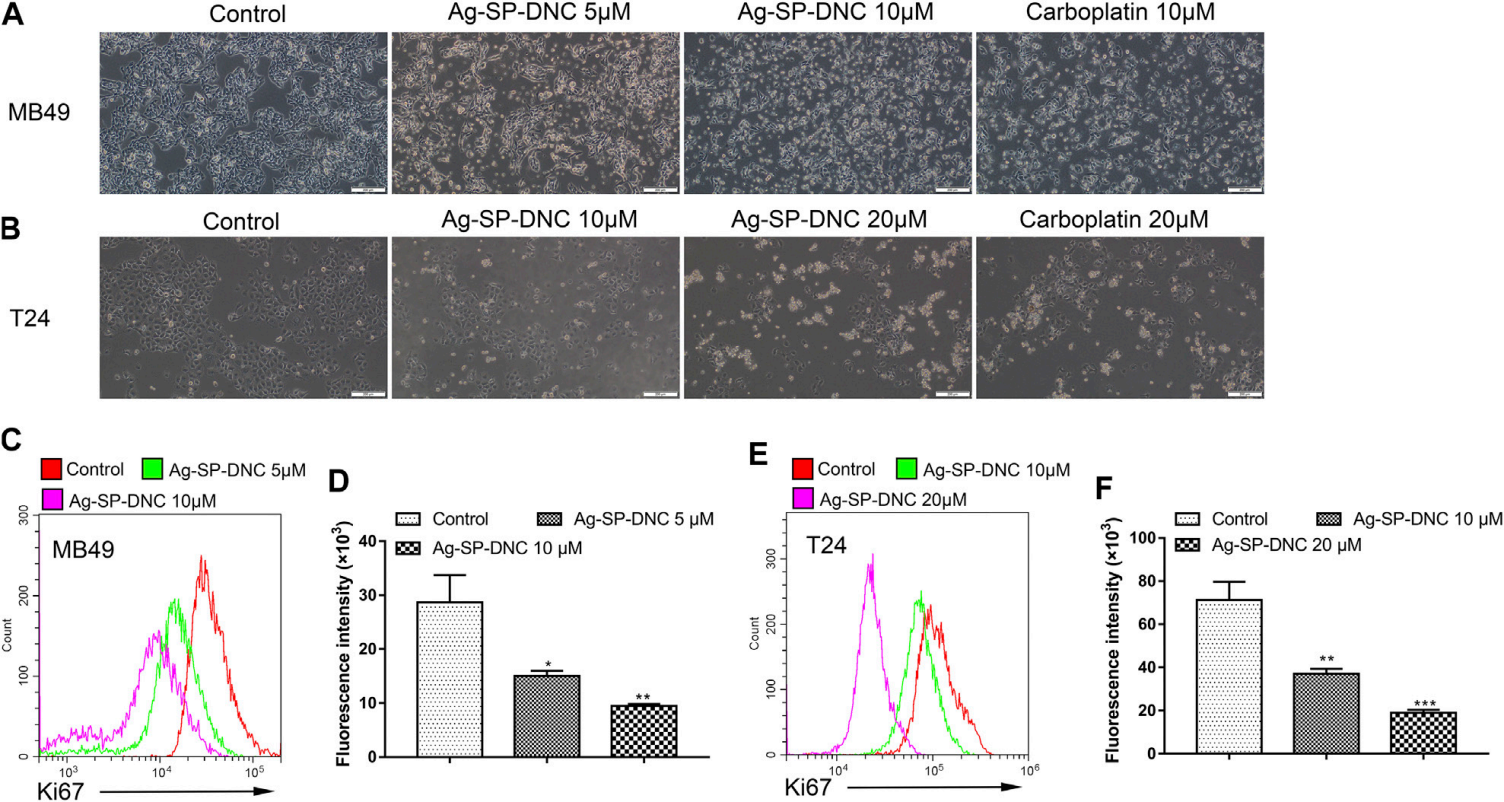
Ag-SP-DNC shows potent growth inhibition in bladder cancer cells *in vitro*. **(A)** Representative images of MB49 treated with Ag-SP-DNC or carboplatin for 48 h. The representative fields were photographed at 100x magnification.**(B)** Representative images of T24 treated with Ag-SP-DNC or carboplatin for 48 h. The fields were photographed at 100x magnification. **(C,D)** Ki67 expression of MB49 cells was measured by flow cytometry. **(C)** Representative histograms were shown. **(D)** Cell associated mean relative fluorescence intensities. Data are mean ± SEM; n = 3. *p < 0.05, **p < 0.01. **(E**,**F)** Ki67 expression of T24 cells was measured by flow cytometry. **(E)** Representative histograms were shown. **(F)** Cell associated mean relative fluorescence intensities. Data are mean ± SEM; n = 3. **p < 0.01, ***p < 0.001.

The effect of Ag-SP-DNC on cell proliferation in bladder cancer cell lines, including MB49 and T24, was evaluated. As shown in Figures 1B,C, it was first confirmed that the MB49 and T24 cells were sensitive to Ag-SP-DNC, with IC50 values of 8.03 and 12.24 μM, respectively. Then, Ag-SP-DNC concentrations for MB49 (5 and 10 μM) and T24 cells (10 and 20 μM) were selected for further experiments. The photos showing the inhibitory status of MB49 and T24 cells were captured by a microscope equipped with a camera. As presented in Figures 2A,B, following Ag-SP-DNC treatment, MB49 and T24 cells displayed a rounded morphology. As shown in Figures 2C–F, Ki67 level analysis by flow cytometry confirmed that Ag-SP-DNC decreased Ki67 expression in bladder cancer cells. In combination, these data suggested that Ag-SP-DNC inhibited bladder cancer cell proliferation and reduced the Ki67 expression levels.

### Ag-SP-DNC Induces G0/G1 Phase Arrest in Bladder Cancer Cells

**FIGURE 3:**
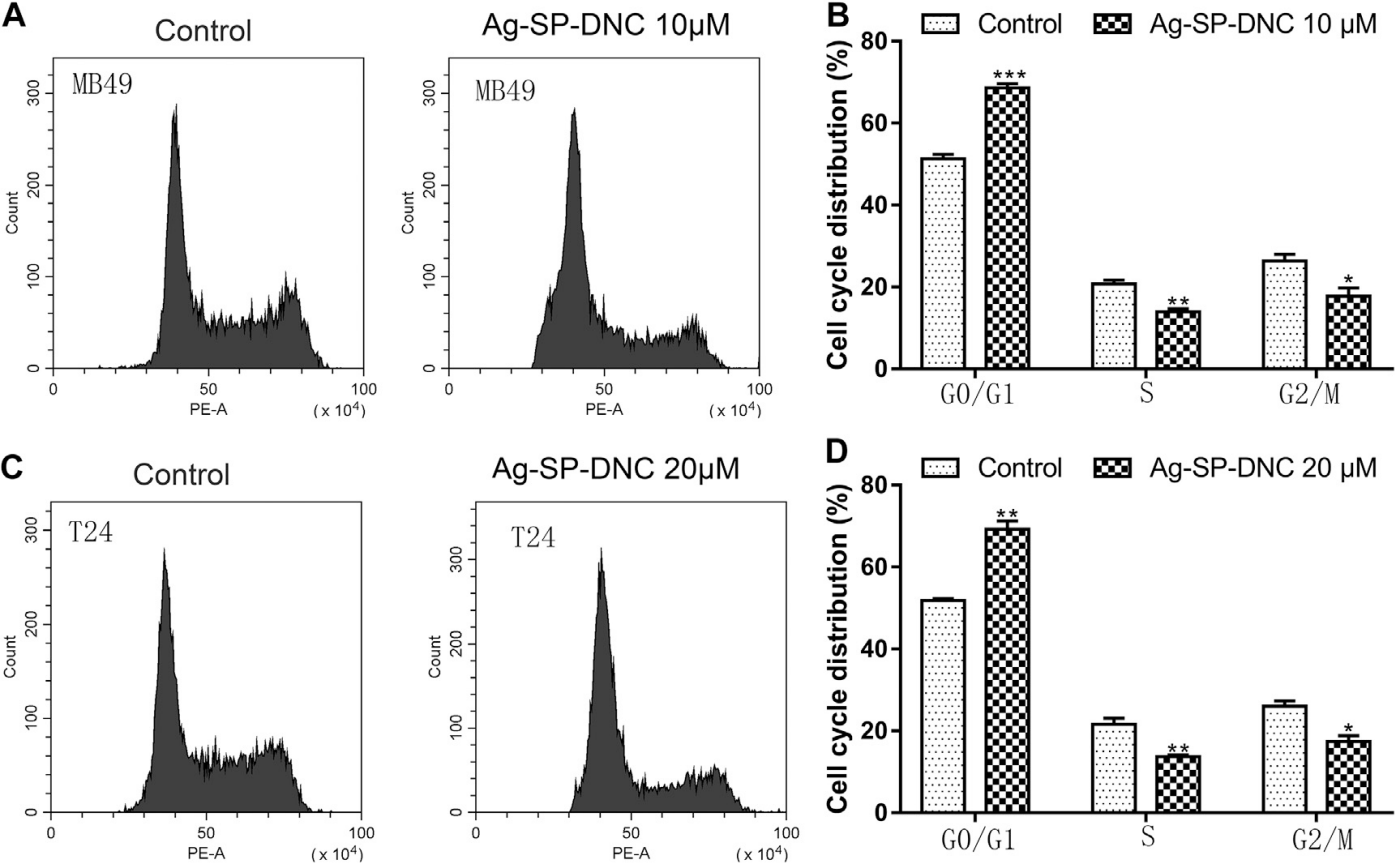
Ag-SP-DNC induces cell cycle arrest at G0/G1 stage in bladder cancer cells. Bladder cancer cells were treated with Ag-SP-DNC for 24 h, then stained with PI and analyzed using flow cytometry. **(A)** A fluorescence pattern of PI stained MB49 cells with or without Ag-SP-DNC treatment. **(B)** The statistical data of cell cycle distribution. Data are mean ± SEM; n = 3. *p < 0.05, **p < 0.01, ***p < 0.001. **(C)** A fluorescence pattern of PI stained T24 cells with or without Ag-SP-DNC treatment. **(D)** The statistical data of cell cycle distribution. Data are mean ± SEM; n = 3. *p < 0.05, **p < 0.01.

Next, flow cytometric analysis was performed to further examine whether Ag-SP-DNC affected the proliferation of bladder cancer cells by altering cell cycle progression. As shown in Figure 3, the proportion of MB49 and T24 cells at the G0/G1 phase increased substantially from 51.27 and 51.80% to 68.65 and 69.26% after 24 h of Ag-SP-DNC treatment, accompanied with a decrease in the S and G2/M phases of the cell cycle. Together, these observations indicated that Ag-SP-DNC inhibited the proliferation of bladder cancer cells by mitigating the G0/G1 transition.

### Ag-SP-DNC Induces Bladder Cancer Cell Apoptosis

**FIGURE 4:**
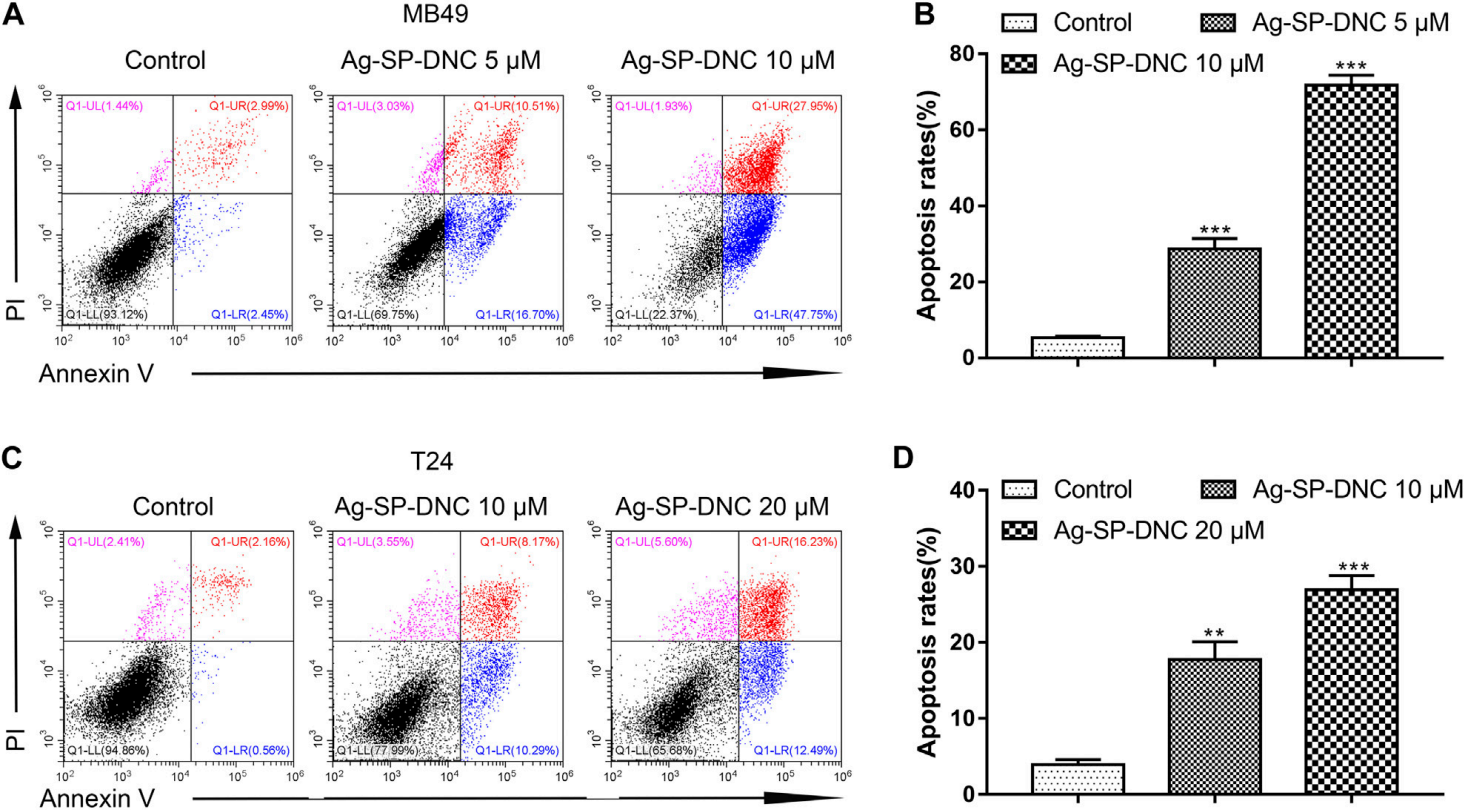
Ag-SP-DNC induces bladder cancer cells apoptosis. The bladder cancer cells were treated with different dose of Ag-SP-DNC for 48 h and then stained with annexin V-FITC/PI followed by flow cytometry analysis. **(A)** The fluorescence pattern of annexin V-FITC and PI stained MB49 cells. **(B)** Percentages of annexin V positive cells for different treatments. Data are mean ± SEM; n = 3. ***p < 0.001. **(C)** The fluorescence pattern of annexin V-FITC and PI stained T24 cells. **(D)** Percentages of annexin V positive cells for different treatments. Data are mean ± SEM; n = 3. **p < 0.01, ***p < 0.001.

To further understand the anticancer mechanisms of Ag-SP-DNC in bladder cancer cells, apoptosis in MB49 and T24 cells was revealed by measuring the Annexin V-FITC/PI staining of apoptotic cells via cytometric analysis. The bladder cancer cells exhibited a significantly dose-responsive increase in apoptotic fractions in Ag-SP-DNC-treated cultures for 48 h, as compared with the control (Figure 4). These observations suggested that Ag-SP-DNC played an anticancer role by inducing apoptosis in bladder cancer cells.

### Ag-SP-DNC Inhibits Tumor Growth in Orthotopic MB49 Bladder Cancer Model

**FIGURE 5:**
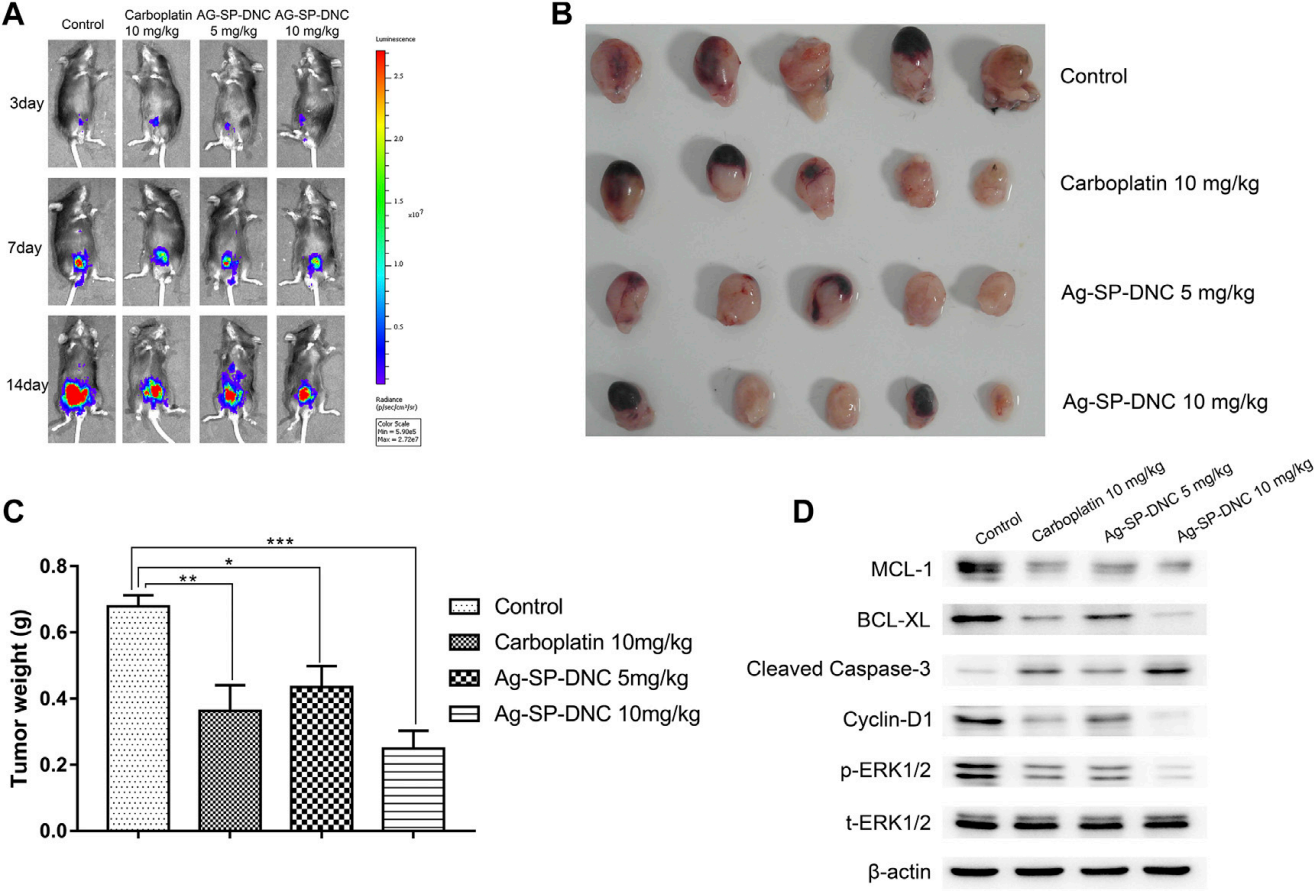
*In vivo* therapy to orthotopic bladder tumors. **(A)** *In vivo* bioluminescence images of MB49 tumor-bearing mice with different treatments. **(B)** Photograph of all collected bladders collected on day 14 post implantation. **(C)** Weight of all collected bladders collected on day 14 post implantation. Data are expressed as the mean ± SEM; n = 5. *p < 0.05, **p < 0.01, ***p < 0.001. **(D)** Western blot of MCL-1, BCL-XL, cleaved caspase-3, Cyclin D1, ERK1/2 phosphorylation and total ERK1/2 levels of MB49 tumors. Tumors were excised and homogenized in lysis buffer on day 14 postimplantation.

To further investigate Ag-SP-DNC efficacy *in vivo*, we used an orthotopic firefly luciferase-expressing MB49 bladder cancer model to observe the therapeutic effect of cancer. Mice were treated with Ag-SP-DNC while carboplatin was used as a positive control after the tumor establishment was confirmed by the presence of bioluminescent signals 3 days after the cell injection. The IVIS Lumina system was used to determine the progression of orthotopic bladder tumors after different treatment. Throughout the study (Figures 5A–C), a marked tumor regression in the orthotopic MB49 bladder cancer model was observed in response to Ag-SP-DNC treatment, while mice in the control treatment arms showed continued tumor growth. To elucidate the intrinsic mechanisms underlying the anticancer effects of Ag-SP-DNC *in vivo*, we sought to explore the signaling pathway involved by western blot analysis. As shown in Figure 5D, the levels of MCL-1 and BCL-XL were decreased and those of cleaved caspase-3 increased in tumor tissues following treatment with Ag-SP-DNC. As compared with the control group, the levels of cyclin D1 and phosphorylation of ERK1/2 were reduced in tumor tissues of mice treated with Ag-SP-DNC (Figure 5D). In combination, these data strongly suggested that Ag-SP-DNC can inhibit MB49 tumor growth by decreasing the expression of apoptosis and cell cycle-related proteins. We also assessed the toxicity of Ag-SP-DNC *in vivo*. Our results from histopathological analysis indicate that no significant histopathological changes were observed in the vital organs (heart, liver, spleen, lung and kidney) after the Ag-SP-DNC administration compared with control group (Supplementary Figure S1). These data suggested that the toxicity of this drug is tolerable.

### Ag-SP-DNC Induces Excess Cellular Levels of ROS in Bladder Cancer Cells

**FIGURE 6:**
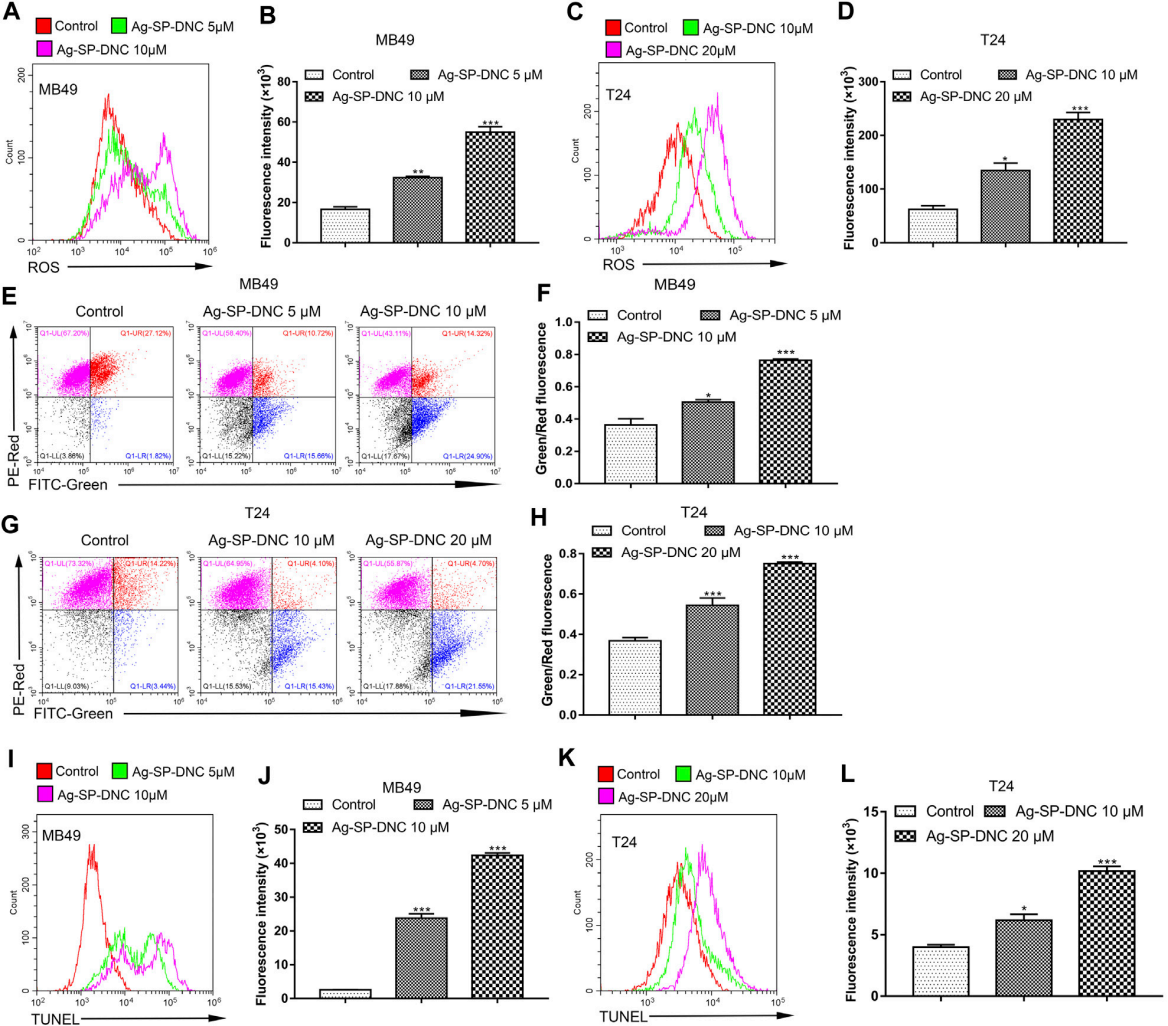
Ag-SP-DNC induces the mitochondrial disruption, ROS production and DNA fragmentation of bladder cancer cells. Bladder cancer cells were treated with Ag-SP-DNC for 24 h. The levels of ROS were measured by DCFH-DA staining and flow cytometric analyses. **(A,B)** ROS levels of MB49 cells were measured by flow cytometry. **(A)** Representative histograms were shown. **(B)** Cell associated mean relative fluorescence intensities. Data are expressed as the mean ± SEM; n = 3. **p < 0.01, ***p < 0.001. **(C,D)** ROS levels of T24 cells were measured by flow cytometry. **(C)** Representative histograms were shown. **(D)** Cell associated mean relative fluorescence intensities. Data are expressed as the mean ± SEM; n = 3. *p < 0.05, ***p < 0.001. Bladder cancer cells were treated with Ag-SP-DNC for 24 h, then harvested and stained with JC-1 followed by flow cytometry analysis. **(E**,**F)** JC-1 staining of MB49 cells were measured by flow cytometry. **(E)** The fluorescence pattern of JC-1 stained MB49 cells. **(F)** Green/Red fluorescence intensities. Data are expressed as the mean ± SEM; n = 3. *p < 0.05, ***p < 0.001. **(G,H)** JC-1 staining of T24 cells were measured by flow cytometry. **(G)** The fluorescence pattern of JC-1 stained T24 cells. **(H)** Green/Red fluorescence intensities. Data are mean ± SEM; n = 3. ***p < 0.001. Bladder cancer cells were treated with Ag-SP-DNC for 24 h, and then harvested and processed by TUNEL staining using flow cytometry analysis. **(I,J)** TUNEL levels of MB49 cells were measured by flow cytometry. **(I)** Representative histograms were shown. **(J)** Cell associated mean relative fluorescence intensities. Data are expressed as the mean ± SEM; n = 3. ***p < 0.001. **(K,L)** TUNEL levels of T24 cells were measured by flow cytometry. **(K)** Representative histograms were shown. **(L)** Cell associated mean relative fluorescence intensities. Data are mean ± SEM; n = 3. *p < 0.05, ***p < 0.001.

ROS accumulation is closely associated with intrinsic apoptotic pathways. The intracellular ROS levels were measured in bladder cancer cells treated with Ag-SP-DNC by detecting the fluorescence of 2,7-dichlorodihydro fluorescent diacetate using flow cytometric analysis. As shown in Figures 6A–D, the results showed that Ag-SP-DNC significantly elevated intracellular ROS levels in a dose-dependent manner. In combination, these data revealed that excess ROS generation is involved in Ag-SP-DNC-induced bladder cancer cell apoptosis.

### Ag-SP-DNC Disrupts the Mitochondrial Membrane Potential of Bladder Cancer Cells

Mitochondria are involved in many activities essential for cell survival, including energy production, calcium homeostasis, redox control, and certain metabolic and biosynthetic pathways. We hypothesized that Ag-SP-DNC induces bladder cancer cell apoptosis through mitochondrial disruption. To pursue this hypothesis, mitochondrial membrane potential analysis was performed in bladder cancer cells treated with Ag-SP-DNC by flow cytometric analysis. As shown in Figures 6E–H, statistical data from flow cytometric analysis suggested that Ag-SP-DNC increased mitochondrial membrane depolarization, which led to an increase in the green/red ratio in JC-1 dye-stained bladder cancer cells. Taken together, these data suggested that Ag-SP-DNC-induced bladder cancer cell apoptosis is closely associated with mitochondrial destruction.

### Ag-SP-DNC Induces Bladder Cancer Cell DNA Degradation

DNA degradation has been widely observed in apoptotic cells. TUNEL assay has been designed to detect apoptotic cells that undergo extensive DNA degradation during the late stages of apoptosis. To evaluate the level of bladder cancer cell DNA degradation with Ag-SP-DNC treatment, the cell TUNEL fluorescence intensity was measured by flow cytometry. As shown in Figures 6I–L, cells treated with Ag-SP-DNC had a high TUNEL fluorescence intensity. These findings indicated that the pro-apoptotic activity of Ag-SP-DNC in bladder cancer cells is connected with internucleosomal DNA degradation.

### Ag-SP-DNC Induces Apoptosis Through a Caspase-dependent Pathway in Bladder Cancer Cells

**FIGURE 7:**
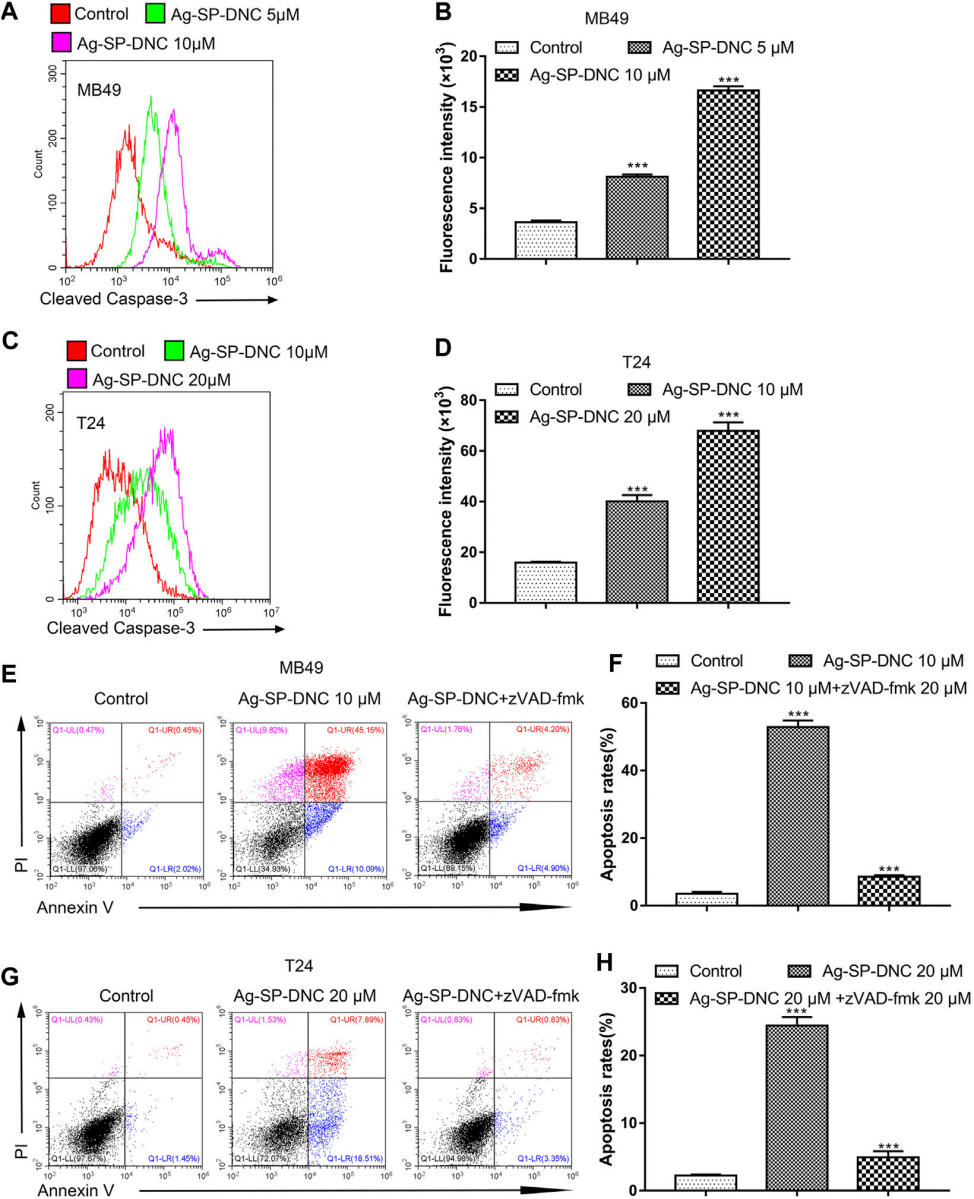
Ag-SP-DNC induces bladder cancer cells apoptosis through cleaved caspase-3. Bladder cancer cells were treated with Ag-SP-DNC for 24 h. The levels of cleaved caspase-3 were measured by flow cytometric analyses. **(A,B)** Cleaved caspase-3 levels of MB49 cells were measured by flow cytometry. **(A)** Representative histograms were shown. **(B)** Cell associated mean relative fluorescence intensities. Data are expressed as the mean ± SEM; n = 3. ***p < 0.001. **(C**,**D)** Cleaved Caspase-3 levels of T24 cells were measured by flow cytometry. **(C)** Representative histograms were shown. **(D)** Cell associated mean relative fluorescence intensities. Data are expressed as the mean ± SEM; n = 3. ***p < 0.001. **(E–H)** The bladder cancer cells were treated with Ag-SP-DNC and caspases inhibitor zVAD-fmk for 48 h, then harvested and stained with Annexin V-FITC and PI followed by flow cytometry analysis. **(E)** The fluorescence pattern of Annexin V-FITC and PI stained MB49 cells. **(F)** Percentages of Annexin V positive cells for different treatments. Data are mean ± SEM; n = 3. ***p < 0.001. **(G)** The fluorescence pattern of Annexin V-FITC and PI stained T24 cells. **(H)** Percentages of Annexin V positive cells for different treatments. Data are mean ± SEM; n = 3. ***p < 0.001.

Apoptosis pathways have been extensively studied, and the activation of caspases is regarded as a crucial characteristic of apoptosis. To assess whether active caspase-3 was correlated with bladder cancer cell apoptosis induced by Ag-SP-DNC, the levels of cleaved caspase-3 were detected by cytometric analysis. As shown in Figures 7A–D, the bladder cancer cells exhibited significantly higher levels of cleaved caspase-3 in Ag-SP-DNC-treated cultures, as compared with control cultures. Bladder cancer cells were then treated with N-benzyloxycarbonyl-valyl-alanyl-aspartyl-fluoromethyl ketone (Z-VAD-FMK), a cell-permeable and irreversible pan-caspase inhibitor (Tao et al., 2020). This substance led to the full inhibition of caspase-3 activation in treated cells. Assuming that Ag-SP-DNC leads to apoptosis execution via caspase-3, Z-VAD-FMK should be as cytoprotective drug. Results showed that caspase inhibition by Z-VAD-FMK leads to the protection of bladder cancer cells from Ag-SP-DNC-induced cell apoptosis (Figures 7E–H). These results demonstrated that the anti-proliferative effect of Ag-SP-DNC in bladder cancer cells is a consequence of apoptosis dependent on caspase-3 activation.

### Ag-SP-DNC Induces Intracellular Free Ca^2+^ Elevation in Bladder Cancer Cells

**FIGURE 8:**
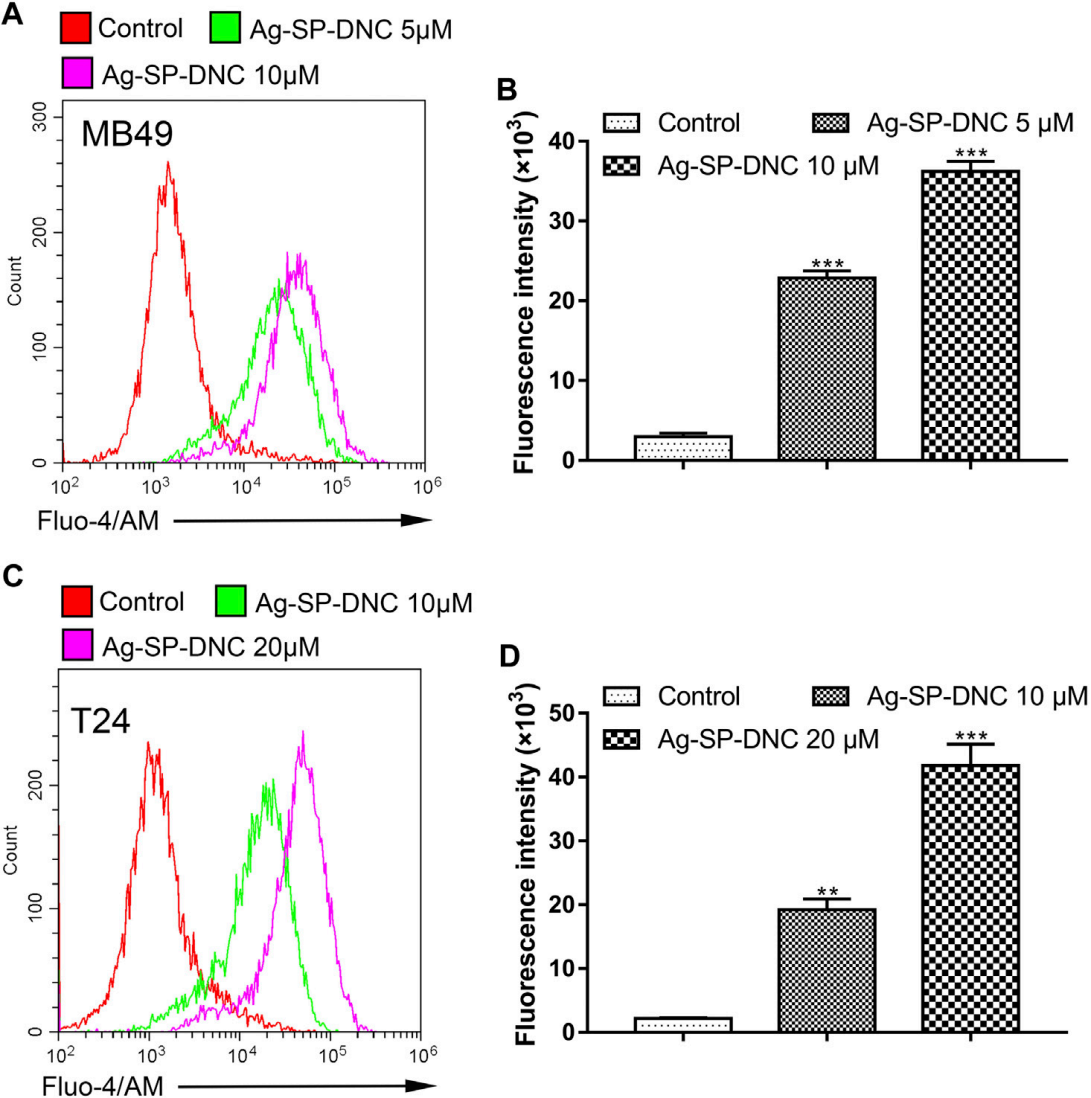
Ag-SP-DNC induces bladder cancer cells intracellular free Ca^2+^ release. Bladder cancer cells were treated with Ag-SP-DNC for 24 h. The levels of intracellular free Ca^2+^ were measured by Fluo-4/AM staining and flow cytometric analyses. **(A**,**B)** Intracellular free Ca^2+^ of MB49 cells were measured by flow cytometry. **(A)** Representative histograms were shown. **(B)** Cell associated mean relative fluorescence intensities. Data are mean ± SEM; n = 3. ***p < 0.001. **(C**,**D)** Intracellular free Ca^2+^ of T24 cells were measured by flow cytometry. **(C)** Representative histograms were shown. **(D)** Cell associated mean relative fluorescence intensities. Data are mean ± SEM; n = 3. **p < 0.01, ***p < 0.001.

Intracellular free calcium (Ca^2+^) is a second messenger molecule and a key element of the cellular response to many abiotic and biotic stresses that regulate various physiological functions in multiple systems. To address whether the regulation of intracellular free Ca^2+^ by Ag-SP-DNC is relevant in bladder cancer cells, flow cytometric analysis was performed by Fluo-4/AM staining. As shown in Figure 8, the bladder cancer cells treated with Ag-SP-DNC exhibited a significantly higher fluorescence intensity in a dose-dependent manner, as compared with the control group. These results showed that Ag-SP-DNC-induced bladder cancer cell apoptosis is associated with the disruption of the intracellular calcium balance.

## Discussion

Bladder cancer is one of the most common genitourinary neoplasms. Although advances in the diagnosis and treatment of bladder cancer have improved patient outcomes, there remains a clear need for effective therapeutic drugs. The present study was designed to evaluate the anticancer efficacy of Ag-SP-DNC and analyze the mechanisms involved in Ag-SP-DNC-induced tumor suppression in a bladder cancer model. Ag-SP-DNC showed an evident cytotoxicity to bladder cancer cells *in vitro* and reduced the bladder tumor burden in tumor-bearing mice. The eukaryotic cell cycle is commonly divided into four stages. The cell cycle progression in normal cells is tightly coordinated and regulated, which is critical for the constant self-renewal, differentiation and homeostasis of the organism system (Matson and Cook, 2017). The fundamental defects in cancer cells are the misregulation of the cell cycle and the loss of the ability to control the growth and division of cells (Senese et al., 2014). The results suggested that cell cycle arrest plays an important role in growth inhibition of bladder cancer cells treated with Ag-SP-DNC.

Apoptosis is a type of programmed cell death and represents a universal efficient cellular suicide pathway. In cancer, one of the therapeutic goals is to trigger tumor-selective apoptosis. The present results showed that Ag-SP-DNC induces apoptosis in bladder cancer cells. Mitochondria are known as the “powerhouses of the cell,” which produce the energy for the cell’s functioning, and play a central role in programmed cell death. In addition, maintenance of the integrity of the mitochondrial membrane plays an important role in cell survival, and mitochondrial dysfunction often leads to cell apoptosis (Sinha et al., 2013). Generally, tumor cells have a higher mitochondrial membrane potential than that of normal epithelial cells. As a consequence, numerous anticancer agents act directly on mitochondria to induce apoptosis, such as drugs that can directly perturb mitochondrial respiration and glycolysis that can lead to an extensive depletion of ATP, which then converge onto the intrinsic death pathway (Kudryavtseva et al., 2016). ROS are a byproduct of cell metabolism within the mitochondria; high concentrations of ROS are often associated with cellular damage, DNA fragmentation and apoptosis. Mitochondria control intracellular ROS levels and interact with different stresses to sustain homeostasis in the cell (Redza-Dutordoir and Averill-Bates, 2016). The outcome of excessive production of ROS is ultimately dependent upon the levels and activities of endogenous antioxidant enzymes (Finkel, 2011; Pandey et al., 2019c). In tumor cells, high levels of ROS are usually associated with necroptotic signaling and cell death.

Calcium, which is an intracellular second messenger, mediates multiple biological processes, including gene transcription, proliferation and cell death. The cytosolic Ca^2+^ is tightly maintained in a very low concentration, and calcium overload can induce apoptosis (Orrenius et al., 2015). Mitochondria uptake Ca^2+^ and are considered a firewall that control Ca^2+^ levels in the cytoplasm. The accumulation of Ca^2+^ in the cytoplasm leads to a rise in mitochondrial ROS generation, causes mitochondrial fragmentation, triggers cytochrome c release and initiates the apoptotic pathway. The critical pathway in apoptosis is the activation of caspases that can be initiated by cytochrome c. Caspase-3 is an effector caspase that recognizes and cleaves short amino acid sequences in many different target proteins, leading to the demise of a cell (Pandey et al., 2019a). The present data revealed that Ag-SP-DNC induces apoptosis in bladder cancer cells through Ca^2+^ and the ROS-mediated mitochondrial pathway.

The Bcl-2 family are vital arbiters in mitochondrial apoptosis (Tait and Green, 2010). Cyclin D1 plays a crucial role in cell cycle regulation, and is constantly overexpressed in cancer through different genomic alterations (Yang et al., 2017). Extracellular signal-regulated protein kinase (ERK)1/2 is a member of the mitogen-activated protein kinase/ERK signaling pathway that plays a critical role in various cellular processes, including cell proliferation, differentiation and survival (Yue et al., 2017). Moreover, ERK1/2 has been described as an important oncogenic factor for bladder cancer. The present data showed that Ag-SP-DNC can inhibit MB49 tumor growth by decreasing the expression of apoptosis and cell cycle-related proteins.

The present study indicated that Ag-SP-DNC had a marked anticancer activity in bladder cancer in an *in vitro* and *in vivo* model by inducing cell cycle arrest and apoptosis in bladder cancer cells. Therefore, Ag-SP-DNC might be considered a potential drug for treatment against bladder cancer. However, intravesical therapy was the most common form of administration for bladder cancer. Further studies are required to explore whether Ag-SP-DNC can be used for intravesical therapy.

## Conclusion

In conclusion, the aim of the study was to clarify the anticancer effects of Ag-SP-DNC in bladder cancer *in vitro* and *in vivo*. Ag-SP-DNC inhibited cell proliferation, increased apoptosis and caused cell cycle arrest in bladder cancer cells *in vitro* and significantly inhibited tumor growth *in vivo*. Ag-SP-DNC could be used as a prospective therapeutic agent for the treatment of bladder cancer.

## Data Availability

The original contributions presented in the study are included in the article/Supplementary Material, further inquiries can be directed to the corresponding author.
