# Supplementary material for: Anticancer Properties and Mechanisms of Singly-Protonated Dehydronorcantharidin Silver Coordination Polymer in a Bladder Cancer Model
Source: Front Pharmacol. 2021 Feb 23;12:618668. doi: 10.3389/fphar.2021.618668 (PMC7940527; doi:10.3389/fphar.2021.618668)
Supplement: Supplementary file 1 [file datasheet1.zip › supplementary materials/Supplementary Materials.docx]

Supplementary Material

**Supplementary Figure**

**
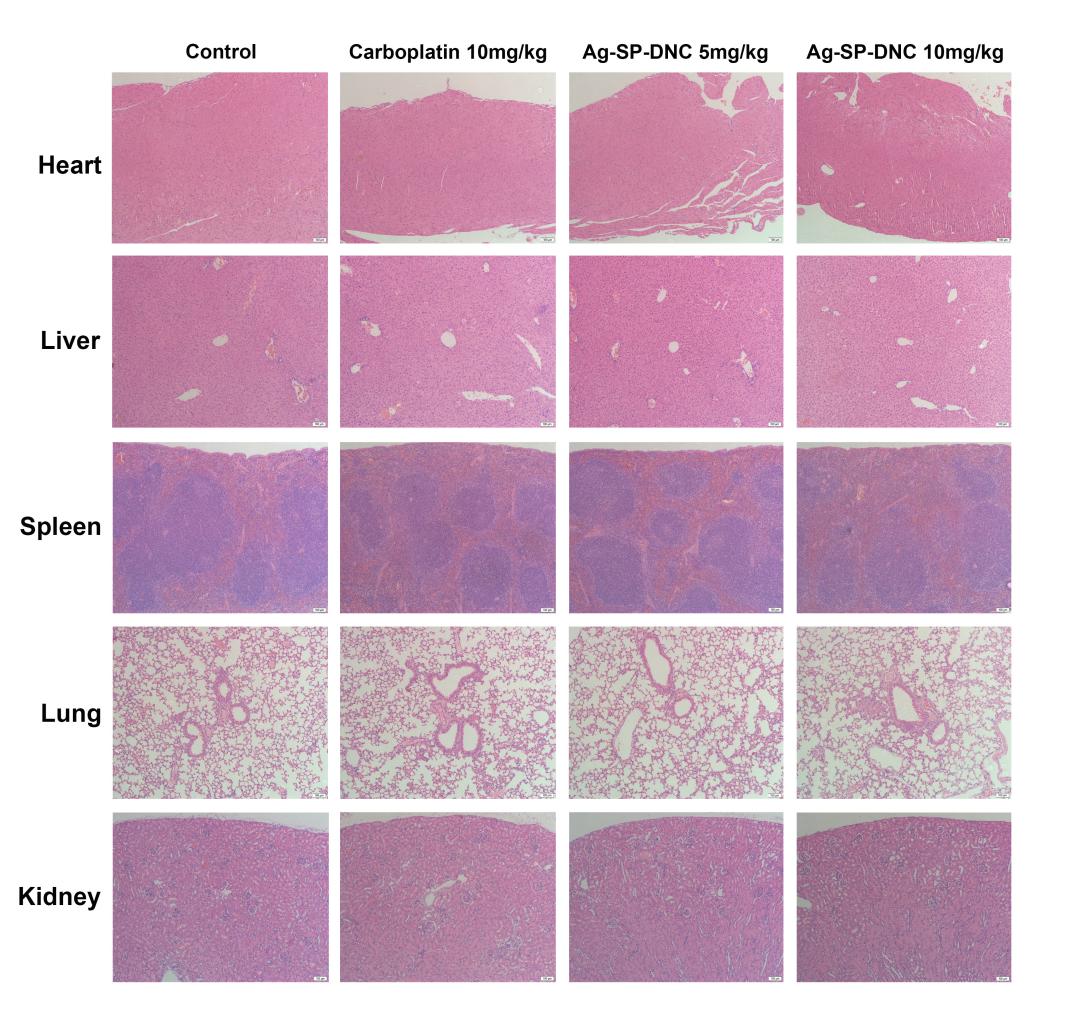
**

**Supplementary figure legend:**

**Histopathology of heart, liver, spleen, lung and kidney tissue from mice treated with Carpoplatin and Ag-SP-DNC, observed under a light microscope.**

Supplementary figure shows the representative organ slices of mice in different groups: control, Carpoplatin (10mg/kg) and Ag-SP-DNC (5 and 10 mg/kg).
