# Supplementary figures and images for: Anticancer Properties and Mechanisms of Singly-Protonated Dehydronorcantharidin Silver Coordination Polymer in a Bladder Cancer Model
Source: Front Pharmacol. 2021 Feb 23;12:618668. doi: 10.3389/fphar.2021.618668 (PMC7940527; doi:10.3389/fphar.2021.618668)

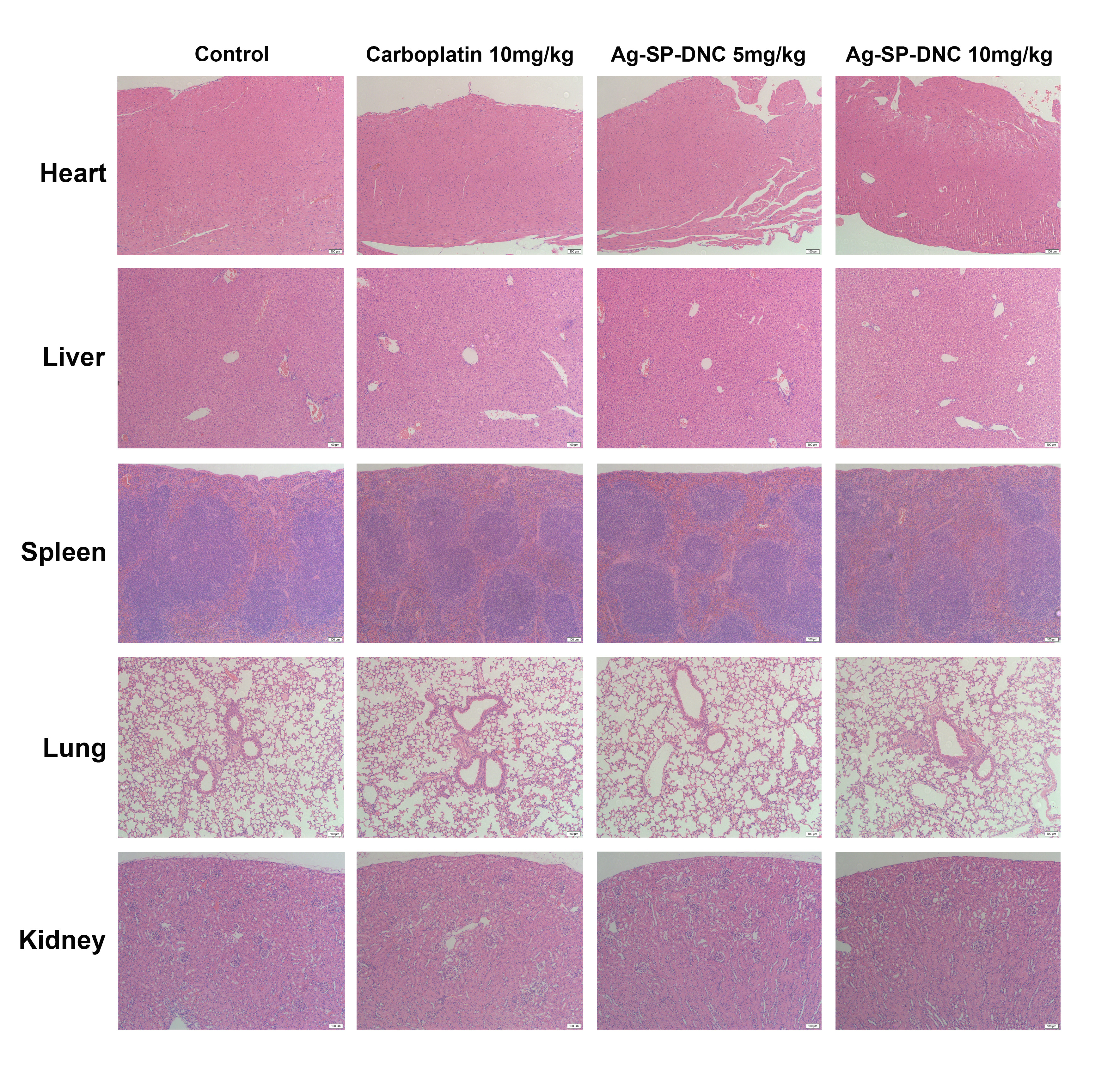

Supplement: Supplementary file 1 [file datasheet1.zip › supplementary materials/supplementary figure.jpg]
